# Two Provincial Workers Join in

**Published:** 1918-10-12

**Authors:** 


*To the Editor of* The Hospital.

### Two Provincial Workers Join In.

*To the Editor of* The Hospital.

Sir,—It would be interesting to learn by what authority "Ierne" takes up the cause of the wrongs of "my nurse," whom she says she knows "as well as Mr. Morris." The nursing profession seems to be roughly divided into two bodies, one which regards nursing chiefly as a means to its own advancement, and the other which has given itself unreservedly to the service of "Its Lords, the Sick," as did the Siennese plague nurses of old. If the former body has authorised "Ierne" to speak for it, well and good, but the members of the latter most certainly have not done so. They realise that the labourer is worthy of his hire, but the hire does not absorb their whole outlook.

It is not true that the whole nursing profession cannot, or dare not, sound the trumpet-note of truth in the board-room, if necessary; but for those timid souls who are afraid there is, as "Ierne" remarks, the refuge of the editor's sanctum to voice their wrongs through the medium of the press. It may be that "the mysterious silence" of which "Ierne" speaks is due to the fact that most nurses at this juncture of the world's history are too much occupied in trying to lighten the burdens of the suffering to trouble much about the righting of their own wrongs, real or imaginary. When there is time to consider these wrongs it should be possible for the righting to be done without the use of pitch.—Yours faithfully,
Two Nurses.

